# 

**Published:** 1851-10

**Authors:** 


The Mississippi Valley Association of Dental Surgeons.—This Association met in Louisville, on 11th, 12th and 13th of last month, and we do not recollect of ever having attended a more interesting meeting of the Society. The discussions were not only interesting but instructive; and, we are sure, if the profession generally felt the importance of such convocations there would be five times as much interest manifested, and instead of some fourteen or fifteen members present, there would be a hundred. We are sorry that a more extended report was not obtained of the discussions. The first two days of the meeting we were quite unwell with a severe headache and took but few notes; indeed the only notes we took were those reported on Dr. Drake's interrogatories, and these present but a brief synopsis of the discussion on these questions.

Our Louisville brethren will be remembered for their kind hospitality, and we hope to see them all next September in the Queen City. We feel assured that the Society will now, not only increase in numbers but in usefulness. It could not have been expected that during the cholera seasons much interest would have been manifested in its meetings.

We hope, at the next meeting, to see many who have not been with us at the last two or three sessions.

American Society of Dental Surgeons.—It was our intention to have given a synopsis of the late proceedings of this Society. This we shall do in our next. We understand the meeting was one of more than ordinary interest.

It would afford us much pleasure to visit Newport, R. I., first Tuesday of next August, and participate in the discussions at the next meeting.

Society of Dental Surgeons of the State of New York.—This Association held an Annual meeting and 'partook of their Annual Supper, at New York, September 9th. We were kindly invited to be present, and we regret we could not avail ourselves of their hospitality. Such meetings will do much to foster and keep up a fraternal feeling, and we hope our New York brethren will enjoy many *Annual Suppers.*

Enlargement of the Register.—We have deterimned hereafter to give in each number of the Register Sixty pages of reading matter instead of Fifty-two; and in addition to this the type used in the editorial department will set up at least one-third more copy, so that in reading matter the Register will be at least one-fifth larger than heretofore. The subscription price will, however, remain the same, and the four volumes already issued will be put to new subscribers (who will pay for volume 5 in advance) at $6.

To our Subscribers.—We shall send with the present number of the Register bills to all who owe for the back numbers, and hope we shall generally meet a response from such. Those living near any of our agents will settle with them, or if they prefer enclose to the editor by mail. It would be a good time also to pay for volume 5. We feel assured our subscribers would not get behind in this matter could they but realize the importance of attending to this matter. We devote much time for their benefit, and we hope they will not let us use our own means to supply them with the latest dental matter.

SEVENTH ANNUAL ANNOUNCEMENT OF

### THE OHIO COLLEGE OF DENTAL SURGERY.

### SESSION OF 1851--'52.

The regular course of Lectures in this Institution commences the first Monday of November and closes on the 20th of February.

FACULTY:

**JAMES TAYLOR, M.D., D.D.S.,**
*Professor of the Principles and Practice of Dental Surgery.*

**THOMAS WOOD, M.D.,**
*Professor of Anatomy amd Physiology.*

**GEORGE MENDENHALL, M.D.,**
*Professor of Pathology and Therapeutics.*

**JOHN ALLEN, D.D.S.**
*Professor of Operative and Mechanical Dentistry.*

**G. L. VAN EMON, A.M., D.D.S.,**
*Lecturer on Dental Chemistry and Demonstrator of Operative and Mechanical Dentistry.*

The arrangements in the laboratory will be such as to afford every facility in mechanical dentistry, and a Demonstrator of acknowledged capacity has been selected for this department.

The following gentlemen have been chosen a committee for the examination of the graduating class:

PHYSICIANS—
Prof. Jesse P. Judkins, M. D., Cincinnati.
" L. M. Lawson, M. D. do.

DENTISTS—
Wm. Wright, M. D., Pittsburgh.
H. R. Smith, D. D. S., Terre Haute, Indiana.
E. Taylor, M. D. D. D. S., Cincinnati.

Tickets for the entire course, including Matriculation fee, $100 00
Diploma fee, - - - - - - - - - - - - 25 00

Good board can be had from $2 to $3 per week.

JAMES TAYLOR, *Dean.*

---

### REMOVAL.

W. Z. REES, Respectfully informs his friends and patrons, that he has REMOVED his Manufactory to Sixth Street, four doors East of Walnut st, North Side. A general assortment of DENTAL AND SURGICAL INSTRUMENTS always on hand and made to Order.

—Also—

A general assortment of **Pearl Handles and Mirrors,** always on hand.

